# Characterization of complete chloroplast genome of *Malus sylvestris* L

**DOI:** 10.1080/23802359.2019.1629352

**Published:** 2019-07-12

**Authors:** Yunfang Xu, Yujie Zhao, Xueqing Zhao, Xuesen Chen, Zhaohe Yuan

**Affiliations:** a Co-Innovation Center for Sustainable Forestry in Southern China, Nanjing Forestry University, Nanjing, China;; b College of Forestry, Nanjing Forestry University, Nanjing, China;; c State Key Laboratory of Crop Biology, Shandong Agricultural University, Tai'an, Shandong, China

**Keywords:** *Malus sylvestris* L, whole genome resequencing, chloroplast genome, phylogenetic analysis

## Abstract

The European wild apple (*Malus sylvestris* L.) is an important economical fruit crop. In this present study, we characterized the complete chloroplast (cp) genome sequence of *Malus sylvestris* L. The complete cp genome is 159,926 bp in length with a typical quadripartite structure, containing a large single-copy region (88,064 bp), a small single-copy region (26,353 bp) and a pair of inverted repeat regions (19,157 bp each). A total of 110 unique genes were found in the newly sequenced genome, including 78 protein-coding genes, 28 tRNA genes, and 4 rRNA genes. Of these, 6 protein-coding genes, 7 tRNA genes, and all 4 rRNA genes are duplicated in the inverted regions. A phylogenetic tree was reconstructed using the neighbor-joining method based on the full length of cp genomes within genus *Malus*. The result showed that *M. sylvestris* L. was clustered together with the cultivated apple. The complete cp genome could provide valuable information for understanding the phylogenetic relationships within the genus *Malus*.

*Malus sylvestris* L., a small tree native to Europe, belongs to the genus *Malus* of the family Rosaceae. It has been identified as one of the main contributors to the domesticated apple *M. domestica* (Ruhsam et al. 2018). In 1986, the tobacco chloroplast genome was first reported (Shinozaki et al. 1986), and subsequently, the number of cp genome sequences has increased due to the rapid development of high-throughput sequencing technologies. The sequencing results are available in public sequence repositories, which are widely used for population genetics, taxonomic, and phylogenetic studies (Lu et al. 2018). Phylogenomic analysis using complete cp genome sequences provides new insight into the evolution and domestication of some important fruit trees (Nikiforova et al. 2013; Jose et al. 2015). In addition, comparing and analyzing cp genomes of related species helps to understand the relationship between the important traits under the control of plastid genome (Yan et al. 2019). In this study, we generated the complete chloroplast genome sequence of *M. sylvestris*, which will be helpful to elucidate the phylogenetic relationship between *M. sylvestris* and other species within genus *Malus*.

Total genomic DNA was extracted from fresh young leaf tissue sampled from Shandong Agricultural University using the DNeasy Plant Mini Kit (Qiagen, Venlo, the Netherlands). The voucher specimen was stored in Shandong Agricultural University (117.112 E, 36.194 N). Illumina paired-end (PE) library was prepared and sequenced using Illumina HiSeq 2500 platform (Illumina, San Diego, CA) using the fastp program to filter the raw paired-end reads (Chen et al. 2018).

Subsequently, a *de novo* assembly was performed using GetOrganelle and applied the reads which are of high quality (Jin et al. 2018). Annotation result was inspected by the online program GeSeq (Tillich et al. 2017) and adjusted manually as needed using Geneious (Kearse et al. 2012). The genome was deposited in the GenBank (MK434923).

The *M. sylvestris* cp genome is 159,888 bp in size. It shows a typical quadripartite structure, including a pair of inverted repeat regions (IRs, 26,347 bp), a large single copy (LSC, 88,035 bp), and a small single copy (SSC, 19,159 bp). In total, 110 unique genes were predicted on this cp genome, including 78 protein-coding, 28 tRNA, and 4 rRNA genes. Six protein-coding genes (*ndhB*, *rpl2*, *rpl23*, *rps12*, *rps7*, *ycf2*), seven tRNA genes (*trnA-UGC*, *trnI-CAU*, *trnI-GAU*, *trnL-CAA*, *trnN-GUU*, *trnR-ACG*, *trnV-GAC*), and all four rRNA genes (*rrn4.5S*, *rrn5S*, *rrn16S*, *rrn23S*) were found to be duplicated. 17 genes contain introns, of which three genes (*clpP*, *rps12*, *ycf3*) exhibit two introns. The overall GC content is 36.58%. The GC content of IRs (42.71%) was higher than LSC (34.26%) and SSC (30.43%).

To resolve the phylogenetic relationships within genus *Malus*, a neighbor-joining (NJ) tree was constructed based on complete cp genome sequences. *Prunus dulcis* and *Prunus serotine* were selected as outgroups. Multiple sequences alignment was performed by HomBlocks. The NJ tree was inferred using MEGA7 based on Kimura 2-parameter model. The result (Figure 1) indicated that *M. sylvestris* was phylogenetically close to *M. domestica*.

**Figure 1.**
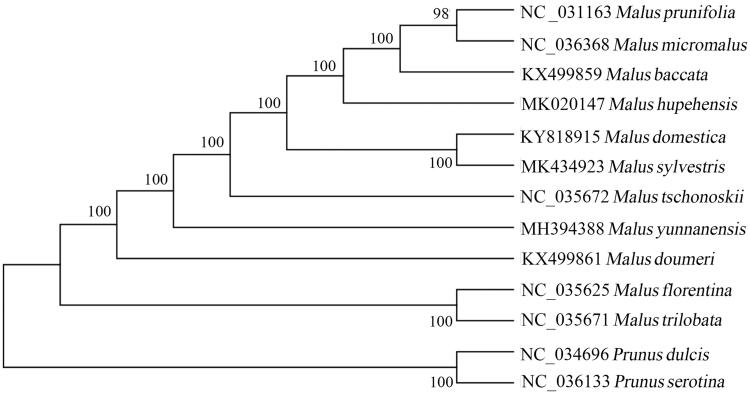
The phylogenetic tree was inferred using the neighbor-joining method. The numbers near each node were bootstrap values inferred from 1000 replicates.
